# An integrated systems biology approach to the study of preterm birth using "-omic" technology - a guideline for research

**DOI:** 10.1186/1471-2393-11-71

**Published:** 2011-10-12

**Authors:** Sara Gracie, Craig Pennell, Gunvor Ekman-Ordeberg, Stephen Lye, James McManaman, Scott Williams, Lyle Palmer, Maureen Kelley, Ram Menon, Michael Gravett

**Affiliations:** 1 Department of Obstetrics and Gynecology, University of Alberta, Edmonton, Alberta, Canada; 2 School of Women's and Infants' Health, The University of Western Australia, Perth, Western Australia, Australia; 3 Division of Obstetrics and Gynecology, Department of Women's and Children's Health, Karolinska University Hospital and Karolinkska Institutet, Stockholm, Sweden; 4 Samuel Lunefeld Research Institute, University of Toronto, Toronto, Ontario, Canada; 5 Department of Obstetrics and Gynecology, Section of Basic Reproductive Sciences, University of Colorado Anschutz Medical Campus, Aurora, Colorado, USA; 6 Center for Human Genetics Research, Vanderbilt University, Nashville, Tennessee, USA; 7 Ontario Institute for Cancer Research Toronto, Ontario, Canada; 8 Department of Pediatrics, University of Washington School of Medicine and Treuman Katz Center for Pediatric Bioethics, Seattle, Washington, USA; 9 Global Alliance to Prevent Prematurity and Stillbirth, an initiative of Seattle Children's, Seattle, Washington, USA; 10 Department of Epidemiology, Emory University, Atlanta, Georgia and The Perinatal Research Center, Nashville, Tennessee, USA; 11 Department of Obstetrics and Gynecology, University of Washington School of Medicine, Seattle, Washington, USA

## Abstract

Preterm birth is the leading cause of neonatal mortality and perinatal morbidity. The etiology of preterm is multi-factorial and still unclear. As evidence increases for a genetic contribution to PTB, so does the need to explore genomics, transcriptomics, proteomics and metabolomics in its study. This review suggests research guidelines for the conduct of high throughput systems biology investigations into preterm birth with the expectation that this will facilitate the sharing of samples and data internationally through consortia, generating the power needed to study preterm birth using integrated "-omics" technologies. The issues to be addressed include: (1) integrated "-omics" approaches, (2) phenotyping, (3) sample collection, (4) data management-integrative databases, (5) international consortia and (6) translational feasibility. This manuscript is the product of discussions initiated by the "-Omics" Working Group at the Preterm Birth International Collaborative Meeting held at the World Health Organization, Geneva, Switzerland in April 2009.

## 

Preterm birth, (PTB - birth before 37 weeks gestation), is the leading cause of neonatal mortality and is associated with up to 75% of long-term morbidity including developmental delay, cerebral palsy, retinopathy of prematurity, and hearing and vision problems [1,2]. Despite medical advances and better understanding of uterine activation and parturition, the rates of PTB have been increasing over the past three decades in developed countries [3]. with current rates ranging from 5-7% [4]. and also complicate 9.6% of all births worldwide [5]. Late PTBs, defined as delivery at 34^+0 ^weeks to 36^+6 ^weeks of pregnancy [6], have risen 25% since 1990, [7]. now accounting for three quarters of preterm deliveries. This stark increase may be attributed to fetal indications, preterm premature rupture of membranes (PPROM) and its associated risks, and the increase in multiple pregnancies associated with assisted reproductive technology [8]

Complicating our understanding of PTB is that it's etiology is multifactorial and varies by gestational age. Among factors associated with increased risk of PTB are maternal smoking during pregnancy, [9,10] advanced maternal age, [11,12] sub-optimal weight gain during pregnancy, [13] maternal stress, [14-16] decidual thrombosis [17], cervical insufficiency [18,19] and the presence of infection [20-22]. In addition, a variety of environmental and genetic play a role in PTB; however the effect size of these factors is not clear. In the United States, PTB occurs disproportionately in women of African ancestry [23,24] even when controlling for social confounders. Twin studies suggest that the heritability of PTB may be 17-36% [25,26]. Clinically, the best predictor of PTB is a prior history, [27,28]. where recurrence risk increases by approximately 15% with each PTB [29]. Further, data suggest that the risk of PTB is inherited across generations [30]. As evidence increases for a genetic contribution to PTB, so does the need to explore genomics, transcriptomics, proteomics and metabolomics in its study.

High throughput systems biology, referred to as "-omics" technology has revolutionized research methodologies. Through these high throughput technologies and the generation of massive data sets, it is now possible to do in an afternoon what previously took several years and yet our understanding of the complex phenotypes of PTB remain incomplete, inconsistent and without clinical clarity. The "-omics" era has seen many publications (> 250, 000) however only a limited number (~6, 000) have been in reproductive medicine (Figure 1). Many of the "-omics" publications relating to PTB have assessed single classes of "-omics" data, utilizing genomics, transcriptomics or proteomics in isolation. The results of many of these "-omics" publications have failed to replicate and their practical value has been limited, failing to translate into clinical practice. The limited successes of singular approaches emphasize the need for integrated approaches to investigate complex phenotypes across "-omics" categories.

**Figure 1:**
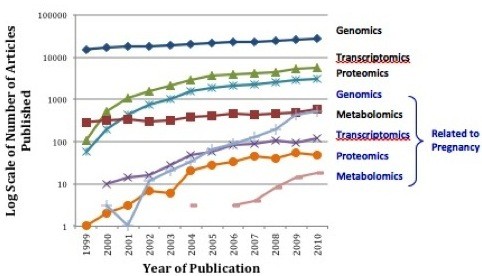
**Systems Biology "-Omics" Publications in Relation to Pregnancy**. Published articles utilizing selected systems biology approaches from 1999-2010. Those related to pregnancy generally less than 3% (note log scale) of the total published articles, and have only begun to increase in 2009. Data abstracted from PubMed with search terms: Human AND English + [transcriptome OR transcriptomics, transcriptome OR transcriptomics + pregnancy, proteome OR proteomics, proteome OR proteomics + pregnancy, genome OR genomics, genome OR genomics + pregnancy, metabolome OR metabolomics, and metabolome OR metabolomics + pregnancy].

To support both singular and integrated systems biology approaches, the "-omics", or systems biology, movement has seen the development of multiple consortiums utilizing high throughput platforms to investigate complex phenotypes. Central to the study of complex phenotypes are accurate phenotype definitions. In the study of PTB, this necessitates collaboration among multiple research groups working synergistically to define phenotypes and to provide adequate sample size [31]Consortia, by design, employ multiple sites for the collection of phenotype data and biological samples with the goal of creating sample sizes large enough to power studies at levels impossible for any single research group, institute or funding opportunity. Moreover, "-omics" technologies require high quality biologic samples with specific, consistent and precise collection and handling. Key to effective consortia is consistency in information gathered, specimen collection, storage and management without which merging of data is problematic.

There is a need for guidelines for the conduct of integrated "-omics" studies into PTB. The genomic, transcriptomic and proteomic working group from the Preterm Birth International Collaborative (PREBIC) meeting in 2009 propose these suggested guidelines. The aim of this article is to establish guidelines for "-omics" studies of PTB such that data and samples collected can be merged, compared and replicated through consortia capable of integrated systems biology methodologies. The issues to be addressed in this guideline include: (1) integrated "-omics" approaches, (2) phenotyping, (3) sample collection, (4) data management-integrative databases, (5) international consortia and (6) translational feasibility.

## Integrated "-omics" Systems Biology Approaches

Until recently the "-omics" era consisted of studies in genomics (the study of genes and their functions), transcriptomics (the study of the complete set of RNA transcripts produced by the genome at one time), and proteomics (the study of the complete set of proteins produced by a species). Recently, through the development of new technologies, metabolomics (the study of small-molecule metabolite profiles generated by cellular processes) has further expanded the "-omics" field. The considerations for using each "-omics" platform in studies of PTB and its sequelae have been reviewed elsewhere [32]. Additionally, the limitations of investigations using "-omics" in isolation have been discussed [33] and emphasize the need for integrated "-omics" approaches as the future path of research.

**Figure 2:**
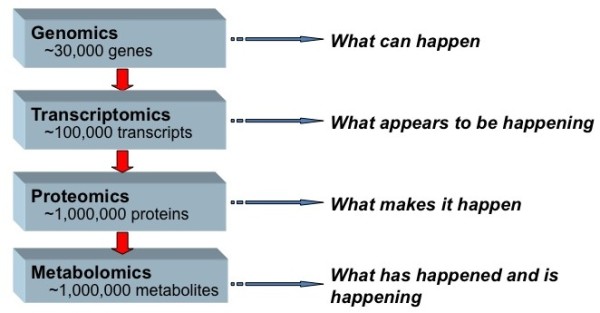
**Systems Biology Tools for Reproductive Medicine**. The main systems biology categories vary in size and physiological information generated from their study. Together, a more complete understanding of PTB pathophysiology can be ascertained. Adapted from [Dettmer K, Aronov PA, Hammock BD. Mass spectrometry-based metabolomics. Mass Spectrom Rev. 2007;26:51-78.]

**Figure 3:**
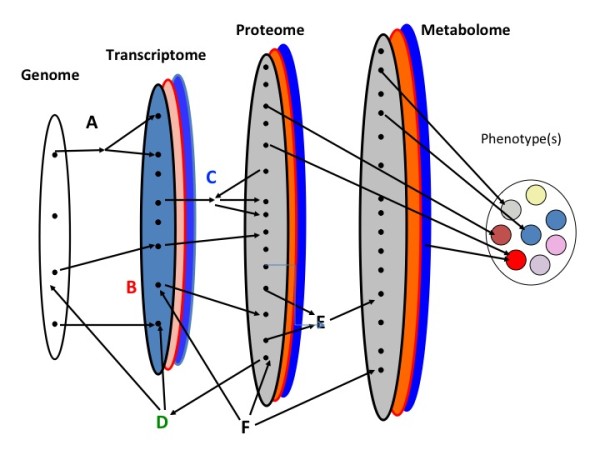
**A General Model of "-Omics" in Complex Disease**. Variation in the genome is represented in the transcriptome which is presented in the proteome. Each level is represented by an oval. For the genome each dot in the oval is a different gene or sequence variant. These variants are expressed as part of the transcriptome. However, unlike the genome which is essentially invariant among cells and tissues the transcriptome can differ substantially. Different tissues are represented by overlapping ovals. Similarly, the transcriptome is translated into the proteome differently in different tissues (again represented as overlapping ovals). The proteome affects the metabolome in a tissue specific manner and the latter two sultimately influence the phenotype. This simple model is modified by multiple factors within and among levels noted on the figure as: A) Differential splicing that can be affected by the proteome; B) siRNA and/or micro RNA; C) post-translation modification of proteins; D) transcription factor binding; E) receptor ligand binding; F) environmentally induced factors such as epigenetic modifications, mutagenesis or modifier of gene expression.

A step-wise integrated approach is central "-omics", yet without strategic implementation, integrating "-omics" fields may be plagued by limitations comparable to the utilization of singular approaches. Each step, or technique, yields distinctly different information (Figure 2) in discovery research. Transcriptomics, not in isolation but rather as an entry point for investigation, presents unique advantages for the study of PTB and perhaps other complex phenotypes alike. Unlike genomics, transcriptomics provides a snapshot of what appears to be happening at a given point in time in a biological sample. Therefore, if patterns are observed which are specific to PTB phenotypes, the functional consequences (protein products) or genetic predisposition (single nucleotide polymorphisms - SNPs) may be ascertained and feedback interactions and processes explored. Proteomics and metabolomics are also promising techniques as analytic steps essential to integrated discovery research studies. These steps are able to build upon the patterns revealed by transcriptomics, as transcriptomes are putative precursors to the actual physiology. However, sample processing requirements and still rapidly evolving technologies pose special challenges in the use of proteomics and metabolomics, as opposed to candidate driven research. In comparison, genomics is limited by the lack of linear associations between genetic variants and complex phenotypes (Figure 3). It holds its intrinsic value in secondary analyses and should also be including in integrated investigations. Utilizing multiple complimentary techniques strategically in integrated studies may reveal the pathophysiological insights and clinical clarity PTB research seeks to discover.

## The Phenotype of Preterm Birth

The World Health Organization defines PTB as "birth before 37 weeks (or 259 days) gestation"[34]. Preterm birth is therefore unique among adverse pregnancy outcomes in that it is defined by a time point and not be specific etiology or pathophysiology. However, as an obstetric syndrome, PTB represents a common end point to a wide variety of clinical conditions that have been classified inconsistently in a number of ways that have included: 1) gestational age at which delivery occurs; 2) clinical presentation resulting in PTB; and 3) putative pathophysiology responsible for PTB. These classification systems are not mutually exclusive, with each of them offering different benefits depending on the scientific or clinical question of interest, but investigators must be clear and consistent in defining the PTB phenotype within the study population.

The most common classification system for PTB is based on gestational age at delivery where cases are classified into strata of extreme prematurity (< 28 weeks gestation), severe prematurity (28-31 weeks gestation), moderate prematurity (32-33 weeks gestation) and near term prematurity (34-37 weeks gestation)[3]. The majority of PTB occurs between 34 and 37 weeks gestation with smaller numbers occurring at lower gestational ages [35]. Unfortunately, accurate assessment of gestational age is not always possible, especially in low and middle-income settings where the burden of disease is high, and resources limited. The last menstrual period is frequently unknown, early dating ultrasound unavailable, and where most births occur home. The need for better tools to accurately assess gestational age is therefore a key research imperative to facilitate "omics" biology in PTB.

Until such tools are available, alternative classification based upon clinical presentation or proposed pathophysiology are more likely to be of value in understanding the genetic and physiological processes that lead to PTB.

**Figure 4:**
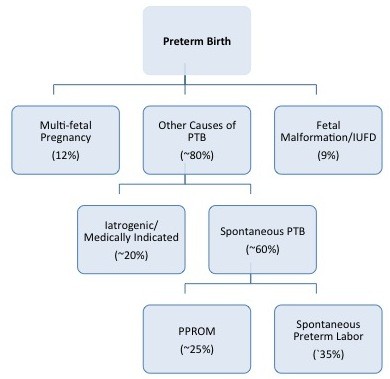
**The Phenotypic Distribution of Preterm Birth**. After excluding multifetal pregnancy and severe fetal malformations/fetal death in-utero, PTB can be classified into two broad clinical pathways - iatrogenic PTB and spontaneous PTB Adapted from Morken NH, Kallen K, Hagberg H, Jacobsson B. Preterm birth in Sweden 1973-2001: rate, subgroups, and effect of changing patterns in multiple births, maternal age and smoking. Acta Obstet Gynecol Scand. 2005;84:558-65 and PENNELL CE, JACOBSSON B, WILLIAMS SM, et al. Genetic epidemiologic studies of preterm birth: guidelines for research. Am J Obstet Gynecol 2007;196:107-18.

In clinical classification schemes, after excluding multi-fetal pregnancy, severe fetal malformations and fetal death in-utero, PTB can be broadly classified into two clinical pathways - iatrogenic PTB and spontaneous PTB (Figure 4).

Iatrogenic, or medically indicated, preterm birth occurs when the benefits to either the mother or fetus of delivery outweigh the benefits of continuing pregnancy. Iatrogenic PTB occurs in about 25% of all PTB with variations from 8.7% to 35.2% according to studied populations [36]. This clinical phenotype includes preeclampsia, diabetes, other maternal medical conditions and fetal growth restriction, a range of conditions with differing etiologies, risk factors and clinical outcomes. As a result of an increased ability to monitor fetal health during pregnancy and recognize the onset of maternal disease earlier, iatrogenic PTB is becoming more common and the increasing rate of late PTB is thought to be largely attributable to iatrogenic causes [3]

Spontaneous preterm birth can result from either spontaneous preterm labor (defined as regular contractions with cervical changes at less than 37 weeks gestation) or preterm pre-labor rupture of membranes (PPROM) defined as spontaneous rupture of the membranes at least 1 hour before the onset of labor and at less than 37 weeks gestation [37]. SPTB accounts for approximately 50% of all PTB (range 23.2% to 64.1%) [36,38]. It is more frequent in populations without any established risk factors in which it represents 50% to 70% of all preterm deliveries [39,40]

It is important to recognize that classifications of PTB based on clinical presentation or gestational age at delivery result in different study groups as the proportion of PTB that are iatrogenic and spontaneous varies between study populations, [41-46] racial backgrounds [41-46] and gestational age [41,47]. Regardless, it is likely that the ultimate pathophysiological mechanisms and pathways involved in PTB are similar across groups.

**Figure 5:**
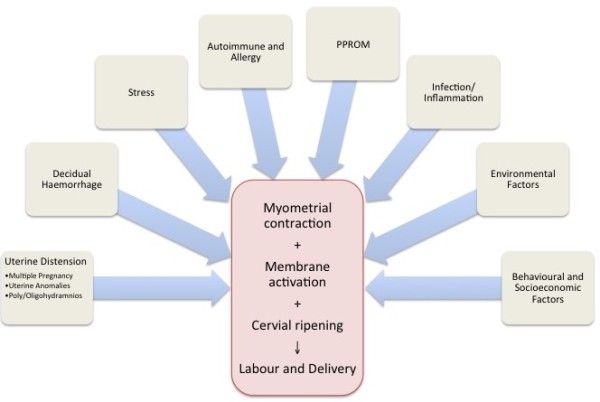
**Proposed Pathophysiological Pathways Leading to Preterm Birth**. The pathophysiology of preterm birth is innately complex and incompletely understood. Several genetic, physiological and environmental factors are associated with preterm birth and contribute to uterine activation, labour and ultimately birth.

It has been hypothesized that PTB and term birth share similar final physiological pathways, but that these pathway are triggered early in PTB [48]. This common pathway of parturition involves the activation of various physiological processes including myometrial contraction, decidual activation, membrane extracellular matrix degradation, weakening and rupture and cervical ripening resulting in labor and delivery [37,48]. While the physiological triggers of this final pathway in term birth are still not well understood, the proposed pathological triggers that have been proposed for PTB are outlined in Figure 5. Activation of one (or more) of these triggers and their interaction with environmental factors and genetic susceptibility in the host can lead to activation of the common parturition pathway at an earlier gestational age and result in PTB.

This complexity is often disregarded in "-omics" research into PTB where spontaneous PTB, preterm premature rupture of membranes, and even iatrogenic PTB are commonly grouped together for analyses. The heterogeneity resulting from grouping known clinical presentations together decreases the sensitivity and power of many research studies. It is vital that PTB is distinguished by its different phenotypes prior to all analyses, including those utilizing "-omics" technologies. Although this may decrease numbers in any given study, it may increase biological homogeneity, thereby potentially replacing the lost statistical power.

## Sample Collection and Processing

Sample collection for "-omics" research is not without it's own considerations, complications and detailed protocols. For PTB research, the biological sample collected is determined by the research question of interest; therefore a wide variety of fluids or tissues such as cervical mucus, blood, urine, saliva, vaginal discharge, myometrium, and uterine tissues are appropriate depending on the investigation. Collection of these specimens should be carefully planned a priori and the consistency of handling closely monitored to assure specimens are representative of the physiology rather than a reflection of ex vivo handling. To ensure samples are of maximum utility to individual and consortia investigations, extracted DNA, RNA, proteins or metabolites should be handled consistently. Table 1 provides an outline of approaches that can be utilized. The availability of biological specimens by itself is not sufficient for integrated "-omics" approaches to PTB research. Detailed documentation of the phenotypes is, as noted above, essential. Regardless of the classification system for PTB employed (Section 2), each sample should have the minimal dataset available (Table 2[41]) and if possible, the optimal dataset (Table 3[41]) described originally for genetic epidemiology studies of PTB but which translate directly into integrated "-omics" approaches in general.

**Table 1:** Sample processing for "-omics" studies of preterm birth

*"-omics" Platform*	*Appropriate Samples*	*Special Considerations*
Genomics (DNA)	Blood Saliva	Stable at room temperature but best if refrigerated Salivette superior to buccal swab for high through-put genotyping

Transcriptomics (RNA)	Blood Amniotic fluid Cervico-vaginal fluid Myometrium Amnion Chorion Decidua	All samples require appropriate collection equipment, meticulous processing and sample storage to insure integrity of RNA for analysis

Proteomics (protein)	Plasma Amniotic fluid Cervico-vaginal fluid Myometrium Amnion Chorion Decidua Urine	All samples require rapid preparation and preservation (+/- protease inhibitors) to prevent non-specific protein degradation

Metabolomics	Blood Amniotic fluid Urine	Samples require rapid metabolic "quenching" (e.g., flash freezing or acid precipitation) to prevent degradation of metabolome

Suggested biological samples and sample handling considerations for each stream of "-omics" technology for the study of preterm birth. The optimal sample to collect should be determined by the driving research question for each investigation.

**Table 2:** The PREBIC Minimal Dataset for "-Omics" Studies on Preterm Birth

*Minimal Dataset*
• Spontaneous initiation vs. Medically indicated preterm birth

• Living fetus vs. intrauterine death at labor initiation

• Singleton vs. multi-fetal gestation

• Gestational age at delivery

• Environmental exposures (tobacco, ethanol, recreational drugs)

• Maternal age

• Parity

• Past obstetrical history

• Ethnicity

The minimal dataset required for international merging of biological samples for "-omics" studies on preterm birth. Adapted from PENNELL CE, JACOBSSON B, WILLIAMS SM, et al. Genetic epidemiologic studies of preterm birth: guidelines for research. Am J Obstet Gynecol 2007;196:107-18.

**Table 3:** Additional Information Desirable for Optimal Dataset for "-Omics" Studies on Preterm Birth

*Optimal Dataset*
• Demographic ○ Socioeconomic status, maternal education

• Clinical ○ Spontaneous onset vs. induced labor

• Maternal ○ Body mass index (BMI), nutritional status, weight gain, uterine anomaly, psychological stress, medications, uterine or cervical surgery, mode of conception, infection, pre-existing medical conditions, pregnancy complications

• Fetal ○ Birth weight, anomalies, infection

• Placental histopathology

• Family history ○ History of preterm birth, maternal gestational age at delivery

The optimal dataset suggested for international merging of biological samples for "-omics" studies on preterm birth. Adapted from PENNELL CE, JACOBSSON B, WILLIAMS SM, et al. Genetic epidemiologic studies of preterm birth: guidelines for research. Am J Obstet Gynecol 2007;196:107-18.

## 4. Data Managements - Integrative Databases

A major limitation to current progress in understanding the genetic predispositions to PTB is that only a limited number of genetic epidemiologic studies are available representing various ethnic/racial groups globally [49]. Therefore, there is a critical need for large and comprehensive clinical resources linked to biospecimen banks. At the level of individual investigators or small teams of researchers, clinical, environmental and biological data are continually being collected for studies with relatively small sample sizes. While it is possible to obtain high quality, mergeable data on large numbers of high-risk pregnancies, the use of this approach is limited (in part) by the absence of field standards and guidelines. Without these navigational beacons, the current use of inadequately sized cohorts or samples has resulted in inconsistent and possibly spurious initial findings for "-omics" results in PTB studies. This is likely due to the multi-genic/multifactorial origin of PTB where any given factor/gene may contribute at most a few percent to the phenotypic variation.

If consistency is present in sample and data collection, an integrated international dataset becomes possible and transparent. The creation of integrated databases that contain both clinical (phenotype) data and biospecimen data has two additional major benefits: access and dissemination. This will allow researchers across the globe to work synergistically to attempt to answer many of the unanswered questions about PTB utilizing adequate sample sizes and the latest developments in technology. Robust technology and analytical infrastructure is imperative to support the vast amounts of data generated.

## 5. International Consortia

PTB is a global problem with increasing rates in developed countries yet the vast majority of cases occur in the developing world (Table 4) [5]. International consortia are therefore needed to bring together resources, experts and data from low, middle and high-income countries to facilitate "-omics" research of PTB and to disseminate results to all who may benefit.

**Table 4:** Regional Variation in Preterm Birth Rates

*Region*	*Preterm Births *	*Preterm Birth Rate (%)*
**World Total**	**12, 870, 000**	**9.6**

*By Economic Development*		

Developed Regions	1, 014, 000	7.5

Less Developed Regions	7, 685, 000	8.8

Least Developed Regions	4, 171, 000	12.5

*By Region*		

Africa	4, 047, 000	11.9

Asia	6, 907, 000	9.1

Latin America	933, 000	8.1

North America	480, 000	10.6

Europe	466, 000	6.2

Oceania (Australia/New Zealand)	20, 000	6.4

The global burden of preterm birth varies by region with the highest rates occurring in developing regions. Adapted from BECK S, WOJDYLA D, SAY L, et al. The worldwide incidence of preterm birth: a systematic review of maternal mortality and morbidity. Bull World Health Organ;88:31-8.

An integrated "-omics" approach for PTB holds the potential for enormous scientific and, ultimately, clinical benefit. The ultimate goal of such research is the improvement of biological understanding leading to prevention, early diagnosis tools, and treatment for PTB and its associated outcomes.

There are currently consortia established to investigate genome wide associations with PTB. All of these consortia are limited in their sample sizes due to the costs of genotyping and it is likely that meta-analyses of these data will be required to make substantial advances in our understanding of the genetic contributions of PTB. Most international consortia have an organized structure including an executive that contains both consortia leadership and members from each of the individual studies or data collection sites contributing data to the consortia. Detailed memorandums of agreement are required between both participating universities and researchers to facilitate smooth working of these consortia.

One example is the Preterm Birth Genome Project, a global consortium to study genetic predisposition in PTB. This consortium includes investigators not only from industrialized nations but also from low and middle-income countries in South America, Eastern Europe, Asia and Africa. The Preterm Birth Genome Project (PGP) was initiated within PREBIC members in September 2007. This consortium includes investigators from four continents and has established a memorandum of agreement to collaborate on GWAS by pooling resources (DNA) and establishing a database of phenotype definitions. The goals of the PGP consortium have been to 1) create a community of researchers to identify PTB susceptibility genes; 2) pool resources from multiple investigators to conduct GWAS across multiple geographic populations including detailed phenotypic and environmental data; 3) to establish a large pool of replication samples; and 4) to enable deep re-sequencing of genes with significant and/or interesting findings in GWAS. This consortium has been highly successful in both collecting resources (> 5000 cases, > 5000 controls) and also funding research into this rapidly evolving field.

A recent consortium is established by PREBIC biomarker working based on a systematic review of SPTB biomarker literature published between 1965-2008. Due to heterogeneities in study designs including above detailed issues of study designs, phenotype definitions and assay variability between different laboratories, no biomarker emerged as a risk predictor. Preterm Birth Biomarker Project (PBP) is setup to address these issues. This study will identify homogeneous studies/samples from around the globe to be tested on a panel of potential PTB biomarkers.

Similarly, further consortia will be required to utilize "-omics" technology and a systems biology approach to study PTB; however, those in existence rarely have adequate samples amenable to multiple "-omics" analyses. We hope that this paper will motivate others to increase the variety of biological samples collected to better address the major hurdles to the study of PTB.

## Translational Feasibility - Barriers and Constraints

Despite the increasing number of publications documenting the utilization of "-omics" technologies for PTB prediction or preterm labor diagnosis, translation into clinical utility is absent despite its continued promise. This apparent gap in knowledge translation reveals both barriers and constraints to applying insights gained from "-omics" investigations of complex diseases. The inconsistencies in defining PTB phenotypes, sample handling methods and environmental variables have, not surprisingly, made reproducibility of study findings nearly impossible. These mixed messages plague the PTB literature and limit the interpretation of "-omics" generated knowledge, hindering translational feasibility. This is of course not unique to PTB.

Genomic analyses of complex traits such as PTB implicitly and explicitly make assumptions regarding the nature of the risks conferred by genetic variants. The most important of these assumptions is that variants in the nucleotide level are linear (or nearly so) in terms of their effects on disease risk. Therefore, one can test for associations between single nucleotide polymorphisms (SNPs) and PTB with the expectation that the role of any given change is transparent to intermediate processes that are included in the central dogma of molecular biology and its correlates (DNA to RNA to protein; Figure 2). Specifically, a gene is transcribed and the mRNA translated in such a way that changes in base pair composition in a gene encoding a critical protein are easily detectable at the phenotypic level. Although this is a very powerful model and in general approximately true (especially for Mendelian disorders), recent research has indicated that this unidirectional process is not universal and many non-linear processes are part of the progression (Figure 4). The failure of the linear model has many implications for the genetic/genomic analyses of PTB. Foremost is the fact that any changes in the primary DNA sequences are not necessarily directly or easily translated into phenotypic changes. Instead, a large number of intermediate processes modulate the effects of DNA variation. Therefore, changes in the DNA may be difficult to detect using a simple association methodology even though they play a key role in disease etiologies. The goal therefore is to more completely model the overall process of gene to phenotype. As a field, we need to recognize that this approach in time will lead to clinical advances to better predict disease and to the design of more effective preventative strategies and treatment. While it is universally accepted that translation is the goal, this is not a realistic deliverable or tangible aim of any single investigation or approach, although this is often implicitly promised.

The now clear need to link data sets from multiple studies is pivotal to the progress of PTB research. However, the goal of integrated international consortia using "-omics" generated data will further complicate translational feasibility if ethical considerations are not addressed proactively at the individual study and consortia levels. When conducting integrated "-omics" investigations ethics boards, participants and researchers face new challenges beyond that of the complexity of the huge data sets produced. The most significant of these include: (1) ensuring robust informed consent and community engagement, (2) dissemination or sharing of research results or other benefits, and (3) sensitivity to the special concerns of participants, typically, pregnant women.

First, consortia will need to address a reoccurring question "how can robust informed consent be sought from participants?"[50-52]. Because the details of future joint ventures are often unknown or impossible to anticipate, obtaining robust informed consent from participants at the time of enrolment to share samples with international consortia is difficult. Similarly, ethics committees face challenges during review, as they cannot evaluate each unanticipated use of data. Participants themselves may be hesitant to partake in studies when the destiny of their samples and information is unknown. The de-identification of biological samples and clinical information is designed to protect the confidentiality of individual participants. However, exactly what information collected by the original study team would be required by a secondary investigator to merge datasets? This may result in only the partial de-identification of participants. Therefore each study design must consider and clarify during the process of informed consent whether consent to share information internationally is optional or required for participation, a choice which may introduce participant bias into study populations. Furthermore, when samples and data are shared internationally, it becomes unclear who bears the onus to maintain the security of the integrated databases and storage of specimens. The security of internationally shared information raises significant ethical and legal considerations.

The second challenge surrounds the dissemination of research findings. Linking and integrating large data sets and their associated biospecimen banks is not inherently straightforward, nor is the dissemination of knowledge generated back to the original communities. These ethical challenges are not unique to PTB research but rather impact biobanking and international consortia efforts in all fields; as such models and lessons developed in relation to cancer research, for example, may be tailored to PTB research. In regards to participants, consortia comprised of representatives from each of the contributing data pools may facilitate the dissemination of study results to their respective participants while still maintaining subject privacy at the consortia level. This would also enable the channeling of information to the original investigators and local communities whom supported the primary data and sample collection. The obligation to return consortia generated results to participants will depend upon the scope and duration of the relationship between the consortia, investigators and participants and therefore may not be possible in all cases. Sharing aggregate consortia generated results may be facilitated by password-protected web-based research updates and newsletters, to keep project level investigators and participants aware of ongoing aggregate findings. These can include contact information for participants and researchers with questions about the studies and aggregate findings. Such sites can also serve as a place for posting educational information about healthy pregnancies, child development or parenting strategies. Because of the psychological burden that attends PTB for women and parents globally, international consortia may be in a position to facilitate social networking among participants or communities by allowing voluntary anonymous participant-participant communication through these websites. This is a way to engage participants in long-term studies and to provide benefit, when significant clinical findings (and therefore direct benefit) for individual participants are not expected.

Third, PTB research also needs to consider the specific expectations and experience of participants [53]. Women or couples who have suffered through one or more preterm births, pregnant women who have experienced a prior preterm or stillbirth, or have a family history of PTB, will likely experience heightened anxiety about their pregnancy that should be taken into consideration during the recruitment process [53]. Similarly, such women may have an expectation that by participating in research, they will "find a cure" to prevent preterm delivery in this pregnancy or a subsequent pregnancy [54]. Attention to these sensitive issues should shape the informed consent process and be considered by consortia utilizing data and specimens collected from these women. As integrated datasets come to the forefront of PTB research, the investments and interests of the participants, local investigators, local communities and international research communities cannot be forgotten. International consortia may be positioned to best preserve these interests by facilitating shared ethical practices rather than leaving each investigator or institution to wrestle with these issues on their own.

## Conclusion

The "-omic"s era presents an exciting time for PTB research. Opportunities now exist to address complex biology utilizing technology that can achieve in a matter of hours what once took many years if possible at all. Although the "-omics" revolution has promise, there are important limitations and constraints to these approaches that cannot go unnoticed. Critical needs at the current time include: 1) improved phenotyping for PTB; 2) large and well-characterized case and control samples with DNA; 3) one or more genome-wide association studies for PTB with broad replication across different populations; and 4) an international consortium for PTB "-omics". Only through the use of multicenter collaborations, careful, detailed phenotyping, specific and consistent sample collections, integrated systems biology approaches and the shedding of simplistic assumptions of the gene-to-phenotype cascade will "-omics" technologies be able to provide new insights into the complex pathophysiology of PTB. The possibilities are within reach and consortia may offer the answer to data management. These guidelines for research provide the direction necessary to harness the promises of "-omics" technologies for advances in the understanding, treatment and prevention of PTB.

## Competing interests

The authors declare that they have no competing interests.

## Authors' contributions

All authors were part of the PREBIC "-Omics" Working Group and together contributed to the conception of the manuscript. SG, CP, SL, JM, SW, LP, MK and MG wrote subsections of the manuscript. SG, CP, SW and MG created or adapted all figures and tables in the manuscript. RM and GEO made substantial contributions to the editing of the manuscript. All authors have read and approved the final manuscript.

## Pre-publication history

The pre-publication history for this paper can be accessed here:

http://www.biomedcentral.com/1471-2393/11/71/prepub

## References

[B1] Huddy CL Johnson A Hope PL Educational and behavioural problems in babies of 32-35 weeks gestation Arch Dis Child Fetal Neonatal Ed 2001 85 1 F23 28 10.1136/fn.85.1.F23 11420317 PMC1721280

[B2] Wang ML Dorer DJ Fleming MP Catlin EA Clinical outcomes of near-term infants Pediatrics 2004 114 2 372 376 10.1542/peds.114.2.372 15286219

[B3] Goldenberg RL Culhane JF Iams JD Romero R Epidemiology and causes of preterm birth Lancet 2008 371 9606 75 84 10.1016/S0140-6736(08)60074-4 18177778 PMC7134569

[B4] Lawn JE Cousens SN Darmstadt GL Bhutta ZA Martines J Paul V Knippenberg R Fogstad H 1 year after The Lancet Neonatal Survival Series--was the call for action heard? Lancet 2006 367 9521 1541 1547 10.1016/S0140-6736(06)68587-5 16679168 PMC7138031

[B5] Beck S Wojdyla D Say L Betran AP Merialdi M Requejo JH Rubens C Menon R Van Look PF The worldwide incidence of preterm birth: a systematic review of maternal mortality and morbidity Bull World Health Organ 88 1 31 38 10.2471/BLT.08.062554 PMC2802437 20428351

[B6] ACOG committee opinion No. 404 April 2008. Late-preterm infants Obstet Gynecol 2008 111 4 1029 1032 10.1097/AOG.0b013e31817327d0 18378769

[B7] Martin JA Kung HC Mathews TJ Hoyert DL Strobino DM Guyer B Sutton SR Annual summary of vital statistics: 2006 Pediatrics 2008 121 4 788 801 10.1542/peds.2007-3753 18381544

[B8] Loftin RW Habli M Snyder CC Cormier CM Lewis DF Defranco EA Late preterm birth Rev Obstet Gynecol 3 1 10 19 PMC2876317 20508778

[B9] Nabet C Ancel PY Burguet A Kaminski M Smoking during pregnancy and preterm birth according to obstetric history: French national perinatal surveys Paediatr Perinat Epidemiol 2005 19 2 88 96 10.1111/j.1365-3016.2005.00639.x 15787882

[B10] Khader YS Al-Akour N Alzubi IM Lataifeh I The Association Between Second Hand Smoke and Low Birth Weight and Preterm Delivery Matern Child Health J10.1007/s10995-010-0599-2 20364365

[B11] Cnattingius S Forman MR Berendes HW Isotalo L Delayed childbearing and risk of adverse perinatal outcome. A population-based study JAMA 1992 268 7 886 890 10.1001/jama.268.7.886 1640617

[B12] Dollberg S Seidman DS Armon Y Stevenson DK Gale R Adverse perinatal outcome in the older primipara J Perinatol 1996 16 2 Pt 1 93 97 8732554

[B13] Viswanathan M Siega-Riz AM Moos MK Deierlein A Mumford S Knaack J Thieda P Lux LJ Lohr KN Outcomes of maternal weight gain Evid Rep Technol Assess (Full Rep) 2008 168 1 223 PMC4781425 18620471

[B14] Copper RL Goldenberg RL Das A Elder N Swain M Norman G Ramsey R Cotroneo P Collins BA Johnson F The preterm prediction study: maternal stress is associated with spontaneous preterm birth at less than thirty-five weeks' gestation. National Institute of Child Health and Human Development Maternal-Fetal Medicine Units Network Am J Obstet Gynecol 1996 175 5 1286 1292 10.1016/S0002-9378(96)70042-X 8942502

[B15] Wadhwa PD Sandman CA Porto M Dunkel-Schetter C Garite TJ The association between prenatal stress and infant birth weight and gestational age at birth: a prospective investigation Am J Obstet Gynecol 1993 169 4 858 865 8238139 10.1016/0002-9378(93)90016-c

[B16] Orr ST Reiter JP Blazer DG James SA Maternal prenatal pregnancy-related anxiety and spontaneous preterm birth in Baltimore, Maryland Psychosom Med 2007 69 6 566 570 10.1097/PSY.0b013e3180cac25d 17636150

[B17] Lockwood CJ Pregnancy-associated changes in the hemostatic system Clin Obstet Gynecol 2006 49 4 836 843 10.1097/01.grf.0000211952.82206.16 17082678

[B18] Iams JD Goldenberg RL Meis PJ Mercer BM Moawad A Das A Thom E McNellis D Copper RL Johnson F The length of the cervix and the risk of spontaneous premature delivery. National Institute of Child Health and Human Development Maternal Fetal Medicine Unit Network N Engl J Med 1996 334 9 567 572 10.1056/NEJM199602293340904 8569824

[B19] Iams JD Johnson FF Sonek J Sachs L Gebauer C Samuels P Cervical competence as a continuum: a study of ultrasonographic cervical length and obstetric performance Am J Obstet Gynecol 1995 172 4 Pt 1 1097 1103 discussion 1104-1096 7726247 10.1016/0002-9378(95)91469-2

[B20] Hay PE Lamont RF Taylor-Robinson D Morgan DJ Ison C Pearson J Abnormal bacterial colonisation of the genital tract and subsequent preterm delivery and late miscarriage BMJ 1994 308 6924 295 298 8124116 10.1136/bmj.308.6924.295 PMC2539287

[B21] Gravett MG Nelson HP DeRouen T Critchlow C Eschenbach DA Holmes KK Independent associations of bacterial vaginosis and Chlamydia trachomatis infection with adverse pregnancy outcome JAMA 1986 256 14 1899 1903 10.1001/jama.256.14.1899 3761496

[B22] Meis PJ Goldenberg RL Mercer B Moawad A Das A McNellis D Johnson F Iams JD Thom E Andrews WW The preterm prediction study: significance of vaginal infections. National Institute of Child Health and Human Development Maternal-Fetal Medicine Units Network Am J Obstet Gynecol 1995 173 4 1231 1235 10.1016/0002-9378(95)91360-2 7485327

[B23] Demissie K Rhoads GG Ananth CV Alexander GR Kramer MS Kogan MD Joseph KS Trends in preterm birth and neonatal mortality among blacks and whites in the United States from 1989 to 1997 Am J Epidemiol 2001 154 4 307 315 10.1093/aje/154.4.307 11495853

[B24] Schempf AH Branum AM Lukacs SL Schoendorf KC The contribution of preterm birth to the Black-White infant mortality gap, 1990 and 2000 Am J Public Health 2007 97 7 1255 1260 10.2105/AJPH.2006.093708 17538050 PMC1913065

[B25] Treloar SA Macones GA Mitchell LE Martin NG Genetic influences on premature parturition in an Australian twin sample Twin Res 2000 3 2 80 82 10.1375/136905200320565526 10918619

[B26] Clausson B Lichtenstein P Cnattingius S Genetic influence on birthweight and gestational length determined by studies in offspring of twins BJOG 2000 107 3 375 381 10.1111/j.1471-0528.2000.tb13234.x 10740335

[B27] Ward K Genetic factors in preterm birth BJOG 2003 110 Suppl 20 117 12763127 10.1016/s1470-0328(03)00061-2

[B28] DeFranco E Teramo K Muglia L Genetic influences on preterm birth Semin Reprod Med 2007 25 1 40 51 10.1055/s-2006-956774 17205422

[B29] Carr-Hill RA Hall MH The repetition of spontaneous preterm labour Br J Obstet Gynaecol 1985 92 9 921 928 10.1111/j.1471-0528.1985.tb03071.x 4041398

[B30] Bhattacharya S Raja EA Mirazo ER Campbell DM Lee AJ Norman JE Inherited predisposition to spontaneous preterm delivery Obstet Gynecol 115 6 1125 1133 10.1097/AOG.0b013e3181dffcdb 20502281

[B31] Biggio J Christiaens I Katz M Menon R Merialdi M Morken NH Pennell C Williams SM A call for an international consortium on the genetics of preterm birth Am J Obstet Gynecol 2008 199 2 95 97 10.1016/j.ajog.2008.06.012 18674654 PMC12358095

[B32] Romero R Espinoza J Gotsch F Kusanovic JP Friel LA Erez O Mazaki-Tovi S Than NG Hassan S Tromp G The use of high-dimensional biology (genomics, transcriptomics, proteomics, and metabolomics) to understand the preterm parturition syndrome BJOG 2006 113 Suppl 3 118 135 17206980 10.1111/j.1471-0528.2006.01150.x PMC7062297

[B33] Ideker T Galitski T Hood L A new approach to decoding life: systems biology Annu Rev Genomics Hum Genet 2001 2 343 372 10.1146/annurev.genom.2.1.343 11701654

[B34] The prevention of perinatal mortality and morbidity. Report of a WHO Expert Committee World Health Organ Tech Rep Ser 1970 457 1 60 4993526

[B35] Laws P Hilder L Australia's mothers and babies 2006. AIHW National Perinatal Statistics Unit Perinatal Statistics 2006 Sydney: AIHW National Perinatal Statistics Unit vol. Cat. no. PER 46

[B36] Moutquin JM Classification and heterogeneity of preterm birth BJOG 2003 110 Suppl 20 30 33 12763108 10.1016/s1470-0328(03)00021-1

[B37] Menon R Spontaneous preterm birth, a clinical dilemma: etiologic, pathophysiologic and genetic heterogeneities and racial disparity Acta Obstet Gynecol Scand 2008 87 6 590 600 10.1080/00016340802005126 18568457

[B38] Mattison DR Damus K Fiore E Petrini J Alter C Preterm delivery: a public health perspective Paediatr Perinat Epidemiol 2001 15 Suppl 2 7 16 11520396 10.1046/j.1365-3016.2001.00004.x

[B39] Morrison JC Preterm birth: a puzzle worth solving Obstet Gynecol 1990 76 1 Suppl 5S 12S 2193277

[B40] Kramer MS Determinants of low birth weight: methodological assessment and meta-analysis Bull World Health Organ 1987 65 5 663 737 3322602 PMC2491072

[B41] Pennell CE Jacobsson B Williams SM Buus RM Muglia LJ Dolan SM Morken NH Ozcelik H Lye SJ Relton C Genetic epidemiologic studies of preterm birth: guidelines for research Am J Obstet Gynecol 2007 196 2 107 118 10.1016/j.ajog.2006.03.109 17306646

[B42] Arias F Tomich P Etiology and outcome of low birth weight and preterm infants Obstet Gynecol 1982 60 3 277 281 7121906

[B43] Main DM Gabbe SG Richardson D Strong S Can preterm deliveries be prevented? Am J Obstet Gynecol 1985 151 7 892 898 3885736 10.1016/0002-9378(85)90667-2

[B44] Piekkala P Kero P Erkkola R Sillanpaa M Perinatal events and neonatal morbidity: an analysis of 5380 cases Early Hum Dev 1986 13 3 249 268 10.1016/0378-3782(86)90060-5 3720612

[B45] Meis PJ Ernest JM Moore ML Causes of low birth weight births in public and private patients Am J Obstet Gynecol 1987 156 5 1165 1168 3578431 10.1016/0002-9378(87)90133-5

[B46] Meis PJ Ernest JM Moore ML Michielutte R Sharp PC Buescher PA Regional program for prevention of premature birth in northwestern North Carolina Am J Obstet Gynecol 1987 157 3 550 556 3631156 10.1016/s0002-9378(87)80005-4

[B47] Morken NH Kallen K Hagberg H Jacobsson B Preterm birth in Sweden 1973-2001: rate, subgroups, and effect of changing patterns in multiple births, maternal age, and smoking Acta Obstet Gynecol Scand 2005 84 6 558 565 15901267 10.1111/j.0001-6349.2005.00765.x

[B48] Romero R Espinoza J Kusanovic JP Gotsch F Hassan S Erez O Chaiworapongsa T Mazor M The preterm parturition syndrome BJOG 2006 113 Suppl 3 17 42 17206962 10.1111/j.1471-0528.2006.01120.x PMC7062298

[B49] Chaudhari BP Plunkett J Ratajczak CK Shen TT DeFranco EA Muglia LJ The genetics of birth timing: insights into a fundamental component of human development Clin Genet 2008 74 6 493 501 10.1111/j.1399-0004.2008.01124.x 19037974

[B50] Haga SB Beskow LM Ethical, legal, and social implications of biobanks for genetics research Adv Genet 2008 60 505 544 18358331 10.1016/S0065-2660(07)00418-X

[B51] Auray-Blais C Patenaude J Biobanking primer: down to basics Science 2007 316 5826 830 10.1126/science.316.5826.830 17495155

[B52] Auray-Blais C Patenaude J A biobank management model applicable to biomedical research BMC Med Ethics 2006 7 E4 10.1186/1472-6939-7-4 16600040 PMC1475589

[B53] Kelley M Rubens CE Global report on preterm birth and stillbirth (6 of 7): ethical considerations BMC Pregnancy Childbirth 10 Suppl 1 S6 10.1186/1471-2393-10-S1-S6 PMC2841776 20233387

[B54] Henderson GE Churchill LR Davis AM Easter MM Grady C Joffe S Kass N King NM Lidz CW Miller FG Clinical trials and medical care: defining the therapeutic misconception PLoS Med 2007 4 11 e324 10.1371/journal.pmed.0040324 18044980 PMC2082641

